# Minimum-Throughput Maximization for Multi-UAV-Enabled Wireless-Powered Communication Networks

**DOI:** 10.3390/s19071491

**Published:** 2019-03-27

**Authors:** Fahui Wu, Dingcheng Yang, Lin Xiao, Laurie Cuthbert

**Affiliations:** 1 Jiangxi Province Key Laboratory of Intelligent Information Systems, Nanchang University, Nanchang 330031, China; fahui97@email.ncu.edu.cn; 2 Information Engineering School, Nanchang University, Nanchang 330031, China; xiaolin@ncu.edu.cn; 3 Information Systems Research Centre, Macao Polytechnic Institute, Macao 999078, China; laurie.cuthbert@isrcmo.org

**Keywords:** UAV communications, minimum-throughput maximization, trajectory design, resource allocation

## Abstract

This paper considers a wireless-powered communication network (WPCN) system that uses multiple unmanned aerial vehicles (UAVs). Ground users (GUs) first harvest energy from a mobile wireless energy transfer (WET) UAV then use the energy to power their information transmission to a data gatherer (DG) UAV. We aim to maximize the minimum throughput for all GUs by jointly optimizing UAV trajectories, and the resource allocation of ET UAV and GUs. Because of the non-convexity of the formulated problem, we propose an alternating optimization algorithm, applying successive convex optimization techniques to solve the problem; the UAV trajectories and resource allocation are alternately optimized in each iteration. Numerical results show the efficiency of the proposed algorithm in different scenarios.

## 1. Introduction

Recently, wireless communications using unmanned aerial vehicles (UAVs) have attracted a great deal of attention because of the mobility and economical nature of UAV systems [1]. Compared with traditional fixed base station communication, UAVs offer many advantages, such as UAV providing an on-demand communication service, and UAVs accommodating themselves to communications circumstances by adjusting their trajectories or deployment position (which usually brings exceptional channel quality).

UAV-enabled communication has been studied in many scenarios. Considering the UAV as a base station, the deployment position was optimized in [2,3] to achieve a balance between coverage area and communication quality. In [4], the UAV was considered to be a mobile relay, and power allocation and the UAV trajectory were jointly optimized. In [5], the wake-up schedule for sensor nodes and UAV trajectory were jointly optimized for a UAV-enabled data collection system. The authors in [6] further studied a two UAVs-enabled data collection system; the user scheduling and trajectory were jointly optimized with UAV difference consideration. In [7], authors investigated the energy trade-off between ground users (GUs) and UAV.

Wireless energy transfer (WET) is a promising solution to prolong the lifetime of low-power rechargeable users [8,9]. Some experimental demonstration of wireless charging smart phones using WET were launched by some startup companies such as Energous [10] and Ossia [11]. Combining UAV with the WET technique, UAV-enabled WET has been studied in [12]. To overcome the “near-far” fairness problem with harvested energy, a successive convex programming-based algorithm was proposed in [12] to design the UAV trajectory. The hover-and-fly trajectory design was proposed in [13] for a UAV-enabled wireless-powered communication network (WPCN) where the ground user harvests energy from the UAV, then uses the harvested energy to power its uplink information transmission to the UAV. To be fair, the trajectory design for two-UAV-enabled WPCN is also investigated in [14]. We did not notice this paper before we finished the first draft; moreover, their consideration is quite ideal due to the assumption of infinite capacity of battery and empty initial energy. It has been verified in [9] that in traditional WPCN the initial energy in the battery and the capacity of the battery would significantly impact the energy use strategy. In this paper, we focus on how the settings of the battery impact the trajectory design and resource allocation for a two-UAV-enabled WPCN, with the safety distance also considered, in our system model, with one UAV being for energy transfer and the other for data gathering. We propose an alternating optimization algorithm jointly optimizing the resource allocation and UAV trajectories.

## 2. System Model

**Figure 1:**
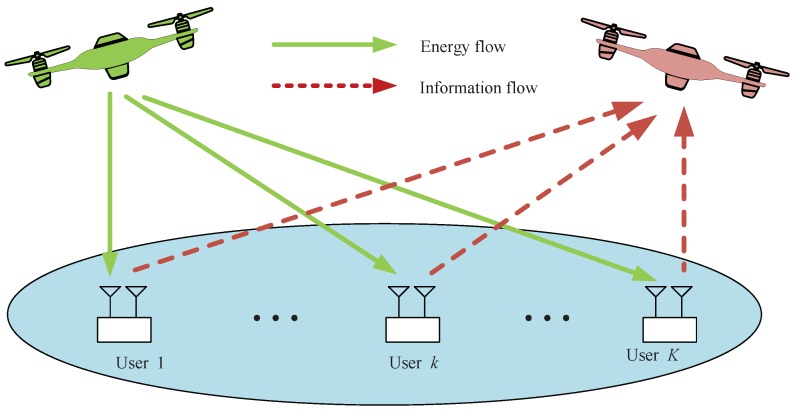
System model.

As shown in Figure 1, we consider a UAV-enabled WPCN with two UAVs and multiple GUs, where the users harvest energy from the energy transfer (ET) UAV and send information to the data gatherer (DG) UAV. All GUs are equipped with two antennas, each of which is dedicated for either energy harvesting or for information transmission, over orthogonal frequency bands. We consider a three-dimensional (3D) Cartesian coordinates system, each user $k\in K≜\left\{1,\ldots ,K\right\}$ is fixed at a given location, denoted by $\left(x_{k},y_{k},0\right)$.

**Figure 2:**
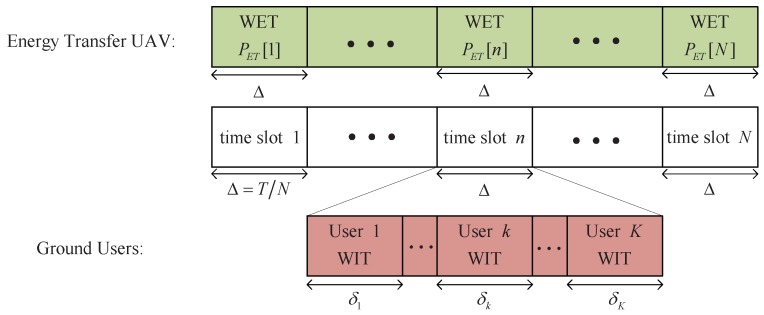
Communication protocol frame for Multi-UAV-Enabled WPCN.

As shown in Figure 2, we focus on UAV-enabled energy/data transmission in one particular time period *T*, which is divided into *N* equal time slots, i.e., T=NΔ. The elemental slot length Δ is chosen to be sufficiently small, such that the distance between the UAVs and the users can be considered approximately constant. We assume that the UAVs fly horizontally at a constant altitude *H*. Let $q_{\mathrm{DG}}\left[n\right]={\left[x_{\mathrm{DG}}\left(n\right),y_{\mathrm{DG}}\left(n\right)\right]}^{T}\in R^{2\times 1}$ and $q_{\mathrm{ET}}\left[n\right]={\left[x_{\mathrm{ET}}\left(n\right),y_{\mathrm{ET}}\left(n\right)\right]}^{T}\in R^{2\times 1}$ denote the trajectory of the ET UAV and DG UAV projected on to the horizontal plane at each time slot $n\in N≜\left\{1,\ldots ,N\right\}$. Accordingly, the distance between user *k* and ET UAV, and the distance from the DG UAV to user *k* are given by:

(1) $$\begin{array}{c} d_{k}^{\mathrm{ET}}\left[n\right]=\sqrt{{∥q_{\mathrm{ET}}\left[n\right]-w_{k}∥}^{2}+H^{2}}\\ d_{k}^{\mathrm{DG}}\left[n\right]=\sqrt{{∥q_{\mathrm{DG}}\left[n\right]-w_{k}∥}^{2}+H^{2}} \end{array}$$

where $w_{k}={\left[x_{k},y_{k}\right]}^{T},k\in K$. The air to ground channel from the UAVs to the GUs is modeled by the free-space-path loss model [4]: the time-varying downlink and uplink channel of user *k* at time slot *n* are given by

(2) $$h_{k}\left[n\right]=\frac{\gamma_{0}}{{∥q_{\mathrm{ET}}\left[n\right]-w_{k}∥}^{2}+H^{2}},k\in K,n\in N$$

and

(3) $$g_{k}\left[n\right]=\frac{\gamma_{0}}{{∥q_{\mathrm{DG}}\left[n\right]-w_{k}∥}^{2}+H^{2}},k\in K,n\in N$$

where $\gamma_{0}$ denotes the channel power at the reference distance d=1m. Each UAV needs to return to its initial location by the end of each period, so the following constraints should be maintained:

(4) $$q_{x}\left[1\right]=q_{x}\left[N\right],x\in \left\{\mathrm{ET},\mathrm{DG}\right\}$$

Considering the maximum speed constraints and collision avoidance constraints, we have:

(5) $$\begin{array}{c} {∥q_{x}\left[n\right]-q_{x}\left[n-1\right]∥}^{2}\leq {\left(\Delta V_{\max}\right)}^{2},n\in \hat{N},x\in \left\{\mathrm{ET},\mathrm{DG}\right\} \end{array}$$

(6) $${∥q_{\mathrm{ET}}\left[n\right]-q_{\mathrm{DG}}\left[n\right]∥}^{2}\geq d_{\min}^{2},n\in N$$

where $\hat{N}=1,\ldots ,N-1$, $V_{\max}$ denotes the maximum speed of UAVs in m/s, and $d_{\min}$ denotes the minimum safe inter-UAV distance in meter. Now, we explain the transmission protocol for our system model: the ET UAV broadcasts the wireless energy signal to all GUs all the time. The transmission power during slot *n* at the ET UAV is denoted by $P_{\mathrm{ET}}\left[n\right]\geq 0$, the total enabled energy for energy transfer at ET UAV is *Q*, i.e., $\sum_{n=1}^{n=N}\Delta P_{\mathrm{ET}}\left[n\right]\leq Q$. If the battery capacity is unlimited, the harvested energy in user *k* at time slot *n* can be given as:

(7) $$E_{k}\left[n\right]=\frac{\gamma_{0}\zeta_{k}\Delta P_{\mathrm{ET}}\left[n\right]}{{∥q_{\mathrm{ET}}\left[n\right]-w_{k}∥}^{2}+H^{2}},n\in N$$

where $\zeta_{k}$ denotes the energy conversion efficiency of user *k*; let $\zeta_{k}=\zeta ,k\in K$ for simplicity.

Each slot is equally divided into *K* subslots for time-division multiple access (TDMA)-based wireless information transmission, the *k*-th subslot of duration $\delta_{k}$ in slot *n* is dedicated to user *k* with transmission power $P_{k}\left[n\right]$. The stored energy of user *k* in the battery at the time instant just before slot *n* is denoted by $B_{k}\left[n\right]$:

(8) $$B_{k}\left[n+1\right]=B_{k}\left[n\right]+E_{k}\left[n\right]-\delta_{k}P_{k}\left[n\right],k\in K,n\in N$$

where $B_{k}\left[1\right]$ denotes the initial energy at the user *k*. The energy for information transmission should be stored before transmission, which leads to energy causality constraints:

(9) $$\sum_{i=1}^{n}\delta_{k}P_{k}\left[i\right]\leq \sum_{i=1}^{n-1}E_{k}\left[i\right]+B_{k}\left[1\right],k\in K,n\in N$$

Suppose the capacity of the rechargeable battery $E_{\max}$ is limited, we have the following constraints:

(10) $$\sum_{i=1}^{n-1}E_{k}\left[i\right]+B_{k}\left[1\right]-\sum_{i=1}^{n}\delta_{k}P_{k}\left[i\right]\leq E_{\max},k\in K,n\in N$$

For all slots, the time resource allocated to user *k* is $\delta_{k}$, i.e., $\sum_{i=1}^{K}\delta_{k}=\Delta$. Accordingly, the average rate $R_{k}$ of user *k* over *N* slots can be written as:

(11) $$\begin{array}{c} R_{k}=\frac{1}{T}\sum_{n=1}^{N}\delta_{k}\log_{2}\left(1+\frac{\frac{\gamma_{0}}{\sigma^{2}}P_{k}\left[n\right]}{{∥q_{\mathrm{DG}}\left[n\right]-w_{k}∥}^{2}+H^{2}}\right) \end{array}$$

Our objective is to maximize the minimum average rate of the users by optimizing both the ET/DG UAV trajectory $q_{\mathrm{ET}}\left[n\right]$ and $q_{\mathrm{DG}}\left[n\right]$, the time allocation $\delta_{k}$, the user power allocation $P_{k}\left[n\right]$ as well as the ET UAV power control $P_{\mathrm{ET}}\left[n\right]$. Defining $R_{\min}=\min_{k\in K}\left\{R_{k}\right\}$, the optimization problem can be formulated as:

$$\begin{array}{c} \left(P1\right)\max_{R_{\min},q_{\mathrm{ET}}\left[n\right],q_{\mathrm{DG}}\left[n\right],\delta_{k},P_{k}\left[n\right],P_{\mathrm{ET}}\left[n\right]}\;R_{\min} \end{array}$$

(12) $$\begin{array}{cc} s.t. & R_{k}\geq R_{\min},k\in K \end{array}$$

(13) $$\begin{array}{c} \sum_{i=1}^{n}\delta_{k}P_{k}\left[i\right]\leq \sum_{i=1}^{n-1}E_{k}\left[i\right]+B_{k}\left[1\right],k\in K,n\in N \end{array}$$

(14) $$\begin{array}{c} \sum_{i=1}^{n-1}E_{k}\left[i\right]+B_{k}\left[1\right]-\sum_{i=1}^{n}\delta_{k}P_{k}\left[i\right]\leq E_{\max},k\in K,n\in N \end{array}$$

(15) $$\begin{array}{c} {∥q_{x}\left[n\right]-q_{x}\left[n-1\right]∥}^{2}\leq {\left(\Delta V_{\max}\right)}^{2},n\in \hat{N},x\in \left\{\mathrm{ET},\mathrm{DG}\right\} \end{array}$$

(16) $$\begin{array}{c} q_{x}\left[N\right]=q_{x}\left[1\right],x\in \left\{\mathrm{ET},\mathrm{DG}\right\}. \end{array}$$

(17) $$\begin{array}{c} {∥q_{\mathrm{ET}}\left[n\right]-q_{\mathrm{DG}}\left[n\right]∥}^{2}\geq d_{\min}^{2},n\in N \end{array}$$

(18) $$\begin{array}{c} \sum_{n=1}^{n=N}\Delta P_{\mathrm{ET}}\left[n\right]\leq Q \end{array}$$

(19) $$\begin{array}{c} 0\leq P_{k}\left[n\right]\leq P_{k}^{\max},0\leq P_{\mathrm{ET}}\left[n\right]\leq P_{\mathrm{ET}}^{\max},n\in N,k\in K \end{array}$$

(20) $$\begin{array}{c} 0\leq \delta_{k}\leq \Delta ,\sum_{k=1}^{K}\delta_{k}=\Delta ,k\in K \end{array}$$

where $P_{k}^{\max}$ and $P_{\mathrm{ET}}^{\max}$ represent the peak transmission power of the user *k* and of the ET UAV, respectively. Due to the constraints in (12)–(14) and (17), the problem is a non-convex optimization problem, which is, therefore, hard to solve with convex optimization techniques. In the following section, we first consider the trajectory optimization with given a resource allocation scheme, then optimize the resource allocation with fixed UAV trajectories.

## 3. Proposed Solution

### 3.1. Trajectory Optimization

For any given recourse allocation $\delta_{k}$, $P_{k}\left[n\right]$ and $P_{\mathrm{ET}}\left[n\right]$, (P1) can be optimized by solving the following problem:

$$\begin{array}{c} \left(P2\right)\max_{R_{\min},q_{\mathrm{ET}}\left[n\right],q_{\mathrm{DG}}\left[n\right]}\;R_{\min}\\ s.t.\;(12)-(17) \end{array}$$

Both $R_{k}$ and $E_{k}\left[n\right]$ are a non-convex function with respect to $q_{\mathrm{ET}}\left[n\right]$ and $q_{\mathrm{DG}}\left[n\right]$, respectively. Please note that ${∥q_{x}\left[n\right]-w_{k}∥}^{2}$, $x\in \left\{\mathrm{ET},\mathrm{DG}\right\}$ is convex with respect to $q_{x}\left[n\right]$; introducing slack variables ${\hat{Q}}_{\mathrm{ET}}\left[k,n\right]={∥q_{\mathrm{ET}}\left[n\right]-w_{k}∥}^{2}$ and ${\hat{Q}}_{\mathrm{DG}}\left[k,n\right]={∥q_{\mathrm{DG}}\left[n\right]-w_{k}∥}^{2}$, (P1) can be reformulated as:

$$\begin{array}{c} \left(P2.1\right)\max_{R_{\min},q_{\mathrm{ET}}\left[n\right],q_{\mathrm{DG}}\left[n\right],{\hat{Q}}_{\mathrm{ET}}\left[k,n\right],{\hat{Q}}_{\mathrm{DG}}\left[k,n\right]}\;R_{\min} \end{array}$$

(21) $$\begin{array}{c} s.t.\;\frac{1}{T}\sum_{n=1}^{N}\delta_{k}\log_{2}\left(1+\frac{\frac{\gamma_{0}}{\sigma^{2}}P_{k}\left[n\right]}{{\hat{Q}}_{\mathrm{DG}}\left[k,n\right]+H^{2}}\right)\geq R_{\min},k\in K \end{array}$$

(22) $$\begin{array}{c} \sum_{i=1}^{n}\delta_{k}P_{k}\left[i\right]\leq \sum_{i=1}^{n-1}\frac{\gamma_{0}\zeta_{k}\Delta P_{\mathrm{ET}}\left[i\right]}{{\hat{Q}}_{\mathrm{ET}}\left[k,i\right]+H^{2}}+B_{k}\left[1\right],k\in K,n\in N \end{array}$$

(23) $$\begin{array}{c} \sum_{i=1}^{n-1}\frac{\gamma_{0}\zeta_{k}\Delta P_{\mathrm{ET}}\left[i\right]}{{\hat{Q}}_{\mathrm{ET}}\left[k,i\right]+H^{2}}+B_{k}\left[1\right]-\sum_{i=1}^{n}\delta_{k}P_{k}\left[i\right]\leq E_{\max},k\in K,n\in N \end{array}$$

(24) $$\begin{array}{c} {\hat{Q}}_{x}\left[k,n\right]\leq {∥q_{x}\left[n\right]-w_{k}∥}^{2},x\in \left\{\mathrm{ET},\mathrm{DG}\right\},k\in K\\ \left(15)–(17\right) \end{array}$$

It can be easily verified that (P2.1) and (P2) have the same optimal solution that is satisfied with equality in constraints (20). Because we can always decrease the distance from the UAVs to the users until they are equal, a larger channel power gain not only brings more harvested energy (which will result in a lager region for data transmission) and the minimum throughput can be increased. However, (P2.1) is still non-convex, so we apply successive convex optimization [15]. Given the trajectory of ET UAV and DG UAV in the *r*-th iteration, due to convex function is lower-bounded by its first-order Taylor expansion at any point [16], in local point $q_{\mathrm{DG}}^{r}\left[n\right]$, $R_{k}$ is lower-bounded by:

(25) $$\begin{array}{cc} R_{k} & =\frac{1}{T}\sum_{n=1}^{N}\delta_{k}\log_{2}\left(1+\frac{\frac{\gamma_{0}}{\sigma^{2}}P_{k}\left[n\right]}{{∥q_{\mathrm{DG}}\left[n\right]-w_{k}∥}^{2}+H^{2}}\right)\\ & \geq \frac{1}{T}\sum_{n=1}^{N}\delta_{k}[\alpha_{\mathrm{DG}}^{r}\left[k,n\right]-\beta_{\mathrm{DG}}^{r}\left[k,n\right]({∥q_{\mathrm{DG}}\left[n\right]-w_{k}∥}^{2}-\theta_{\mathrm{DG}}^{r}\left[k,n])\right]\\ & ≜R_{k}^{\mathrm{lb}} \end{array}$$

where

(26) $$\alpha_{\mathrm{DG}}^{r}\left[k,n\right]=\log_{2}\left(1+\frac{\frac{\gamma_{0}}{\sigma^{2}}P_{k}\left[n\right]}{{∥Q_{\mathrm{DG}}^{r}\left[n\right]-w_{k}∥}^{2}+H^{2}}\right)$$

(27) $$\beta_{\mathrm{DG}}^{r}\left[k,n\right]=\frac{\frac{\gamma_{0}}{\sigma^{2}\ln2}P_{k}\left[n\right]}{\left(\theta_{\mathrm{DG}}^{r}\left[k,n\right]+H^{2}\right)\left(\theta_{\mathrm{DG}}^{r}\left[k,n\right]+H^{2}+\frac{\gamma_{0}}{\sigma^{2}}P_{k}\left[n\right]\right)}$$

Similarly, with given local point $Q_{\mathrm{ET}}^{r}\left[n\right]$, the lower bound for $E_{k}\left[n\right]$ can be expressed as follows:

(28) $$\begin{array}{cc} E_{k}\left[n\right] & =\frac{\zeta_{k}\Delta \gamma_{0}P_{\mathrm{ET}}\left[n\right]}{{∥q_{\mathrm{ET}}\left[n\right]-w_{k}∥}^{2}+H^{2}}\\ & \geq \frac{\zeta_{k}\Delta \gamma_{0}P_{\mathrm{ET}}\left[n\right]}{{∥Q_{\mathrm{ET}}^{r}\left[n\right]-w_{k}∥}^{2}+H^{2}}-\frac{{∥q_{\mathrm{ET}}\left[n\right]-w_{k}∥}^{2}-{∥Q_{\mathrm{ET}}^{r}\left[n\right]-w_{k}∥}^{2}}{{\left({∥Q_{\mathrm{ET}}^{r}\left[n\right]-w_{k}∥}^{2}+H^{2}\right)}^{2}}\\ & ≜E_{k}^{\mathrm{lb}}\left[n\right] \end{array}.$$

Moreover, by applying the first-order Taylor expansion at the given point $Q_{x}^{r}\left[n\right]$ to ${∥q_{x}\left[n\right]-w_{k}∥}^{2},x\in \left\{\mathrm{ET},\mathrm{DG}\right\}$, we have:

(29) $$\begin{array}{cc} {∥q_{x}\left[n\right]-w_{k}∥}^{2} & \geq {∥Q_{x}^{r}\left[n\right]-w_{k}∥}^{2}+2{\left(q_{x}\left[n\right]-w_{k}\right)}^{T}\left(q_{x}\left[n\right]-w_{k}\right) \end{array}$$

For constraints (16), we have the following inequality:

(30) $$\begin{array}{c} {∥q_{\mathrm{ET}}\left[n\right]-q_{\mathrm{DG}}\left[n\right]∥}^{2}\geq -{∥Q_{\mathrm{ET}}^{r}\left[n\right]-Q_{\mathrm{DG}}^{r}\left[n\right]∥}^{2}+2{\left(Q_{\mathrm{ET}}^{r}\left[n\right]-Q_{\mathrm{DG}}^{r}\left[n\right]\right)}^{T}\left(q_{\mathrm{ET}}\left[n\right]-q_{\mathrm{DG}}\left[n\right]\right) \end{array}$$

With given $Q_{x}^{r}\left[n\right],x\in \left\{\mathrm{DG},\mathrm{ET}\right\}$, the problem can be approximated as:

$$\begin{array}{c} \left(P2.2\right)\max_{R_{\min},q_{\mathrm{ET}}\left[n\right],q_{\mathrm{DG}}\left[n\right],{\hat{Q}}_{\mathrm{ET}}\left[k,n\right],{\hat{Q}}_{\mathrm{DG}}\left[k,n\right]}\;R_{\min} \end{array}$$

(31) $$\begin{array}{c} s.t.\;R_{k}^{\mathrm{lb}}\geq R_{\min},k\in K \end{array}$$

(32) $$\begin{array}{c} \sum_{i=1}^{n}\delta_{k}P_{k}\left[i\right]\leq \sum_{i=1}^{n-1}E_{k}^{\mathrm{lb}}\left[i\right]+B_{k}\left[1\right],k\in K,n\in N \end{array}$$

(33) $$\begin{array}{c} \sum_{i=1}^{n-1}E_{k}^{\mathrm{lb}}\left[i\right]+B_{k}\left[1\right]-\sum_{i=1}^{n}\delta_{k}P_{k}\left[i\right]\leq E_{\max},k\in K,n\in N\\ {\hat{Q}}_{x}\left[k,n\right]\leq {∥Q_{x}^{r}\left[n\right]-w_{k}∥}^{2}+2{\left(Q_{x}^{r}\left[n\right]-w_{k}\right)}^{T}\left(q_{x}\left[n\right]-Q_{x}^{r}\left[n\right]\right),x\in \left\{\mathrm{ET},\mathrm{DG}\right\},k\in K,n\in N \end{array}$$

(34) $$\begin{array}{c} d_{\min}^{2}\leq -{∥Q_{\mathrm{ET}}^{r}\left[n\right]-Q_{\mathrm{DG}}^{r}\left[n\right]∥}^{2}+2{\left(Q_{\mathrm{ET}}^{r}\left[n\right]-Q_{\mathrm{DG}}^{r}\left[n\right]\right)}^{T}\left(q_{\mathrm{ET}}\left[n\right]-q_{\mathrm{DG}}\left[n\right]\right),n\in N\\ \left(15)–(16\right) \end{array}$$

(P2.2) is a convex optimization problem that can be solved by existing convex solvers, e.g., CVX [17]. The optimal solution of (P2.2) is the lower bound of (P2.1).

### 3.2. Recourse allocation for ET UAV and Users

With a given UAV trajectory $\left\{q_{\mathrm{ET}}\left[n\right],q_{\mathrm{ET}}\left[n\right]\right\}$ and time allocation $\delta_{k}$, the power allocation can be optimized by:

$$\begin{array}{c} \left(P3\right)\max_{R_{\min},P_{k}\left[n\right],P_{\mathrm{ET}}\left[n\right]}\;R_{\min} \end{array}$$

(35) $$\begin{array}{c} s.t.\;R_{k}\geq R_{\min},k\in K \end{array}$$

(36) $$\begin{array}{c} \sum_{i=1}^{n}\delta_{k}P_{k}\left[i\right]\leq \sum_{i=1}^{n-1}E_{k}\left[i\right]+B_{k}\left[1\right],k\in K,n\in N \end{array}$$

(37) $$\begin{array}{c} \sum_{i=1}^{n-1}E_{k}\left[i\right]+B_{k}\left[1\right]-\sum_{i=1}^{n}\delta_{k}P_{k}\left[i\right]\leq E_{\max}\\ \left(18),(19\right) \end{array}$$

It can be easily checked that (P3) is a convex optimization problem that can be solved efficiently by an optimization tool. Please note that (P3) is a typical energy allocation problem in WPCN, thus the optimal power allocation schemes of each ground user are given by the classic “staircase” water-filling solution. Finally, we need to optimize the time allocation $\delta_{k}$ for given trajectories and power allocation; this is a very easy convex problem and details are omitted here for brevity. Please note that for every given trajectory, we should alternately optimize the power allocation and time resource until convergence. Algorithm 1 can be summarized as follows: 

**Algorithm 1:** Minimum-throughput Maximization Algorithm
1:initialize: $\delta_{k}^{r}=\Delta /K$, given $Q_{\mathrm{ET}}^{r}\left[n\right]$, and $Q_{\mathrm{ET}}^{r}\left[n\right]$, let r=02:
**repeat**
3: **repeat**4:  Solve (P3) by CVX for given $\delta_{k}^{r}$, $Q_{\mathrm{ET}}^{r}\left[n\right]$, and $Q_{\mathrm{ET}}^{r}\left[n\right]$, the optimal solution denote as $P_{k}^{r}\left[n\right]$, $P_{\mathrm{ET}}^{r}\left[n\right]$.5:  Solve time allocation for given $P_{k}^{r}\left[n\right]$, $P_{\mathrm{ET}}^{r}\left[n\right]$, $Q_{\mathrm{ET}}^{r}\left[n\right]$ and $Q_{\mathrm{DG}}^{r}\left[n\right]$, the optimal solution denote as $\delta_{k}^{r}$.6: **until** convergence or maximum number of iterations7: Output $\delta_{k}^{r+1}=\delta_{k}^{r}$, $P_{k}^{r+1}\left[n\right]=P_{k}^{r}\left[n\right]$, and $P_{\mathrm{ET}}^{r+1}\left[n\right]=P_{k}^{r}\left[n\right]$8: Solve (P2.2) by CVX for given $\delta_{k}^{r+1}$, $P_{k}^{r+1}\left[n\right]$, $P_{\mathrm{ET}}^{r+1}\left[n\right]$, $Q_{\mathrm{ET}}^{r}\left[n\right]$ and $Q_{\mathrm{ET}}^{r}\left[n\right]$, the optimal solution is denoted as $Q_{\mathrm{ET}}^{r+1}\left[n\right]$ and $Q_{\mathrm{ET}}^{r+1}\left[n\right]$9: Update r=r+110:**until** converge or maximum number of iterations;


Consider an optional initial trajectory which is formed by connecting the users who locate in the edge area. All users should locate in the area that projects the trajectory on to ground. In this paper, such a trajectory is formed by connecting four users located in four corners. Suppose the total traveling distance of this initial trajectory is $L_{1}$, if $V_{max}T\geq L_{1}$, we choose this trajectory as initial trajectory. Otherwise, the trajectory is initialized as follows. The initial trajectory is a circle where the UAV flies with a constant speed $0\leq V\leq V_{\max}$. The radius of initial trajectory of DG UAV is set as $r_{\mathrm{DG}}^{\mathrm{initial}}=\min\left[\frac{V_{\max}T}{2\pi },\max\left(d_{k}\right)\right]$, where $d_{k}$ denote the distance between user *k* and the geometric center of all users. The radius of the initial trajectory of DG UAV is set as $r_{\mathrm{ET}}^{\mathrm{initial}}=r_{\mathrm{DG}}^{\mathrm{initial}}-d_{\min}$.

## 4. Simulation Results

**Figure 3:**
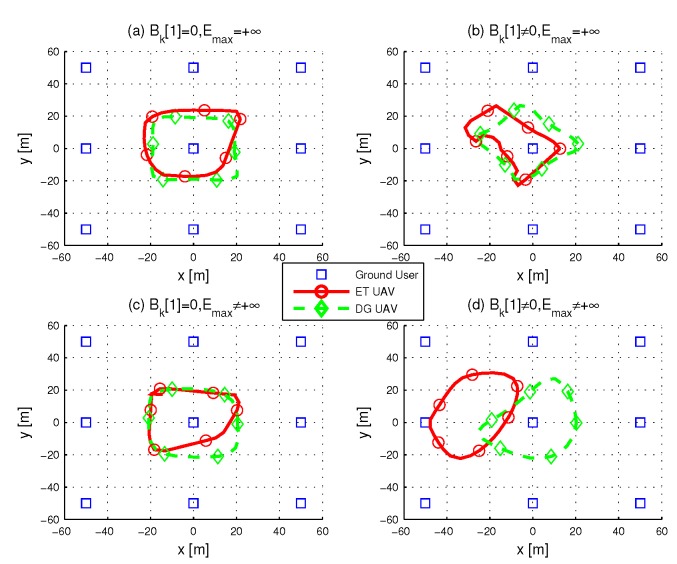
Optimized UAV trajectories when T=30s.

In this section, we provide numerical results to evaluate the performance of our proposed Algorithm 1. Consider a system with K=9 GUs which are in a $100\times 100\;m^{2}$ area, as shown in Figure 3. Similar to [13], the peak transmission power of ET UAV and ground user is set as $P_{\mathrm{ET}}^{\max}=20\;W$ and $P_{k}^{\max}=-10\;\mathrm{dBm}$, respectively. The reference channel gain $\gamma_{0}$ is assumed to $\gamma_{0}=-30\;\mathrm{dB}$ and the receiver noise power is given by σ=−90dBm. The energy harvesting efficiency is set as $\zeta_{k}=0.55$. All the UAVs are assumed to fly at altitude H=10m with maximum speed $V_{\max}=5\;m/s$, and the safety distance is $d_{\min}=5\;m$.

**Figure 4:**
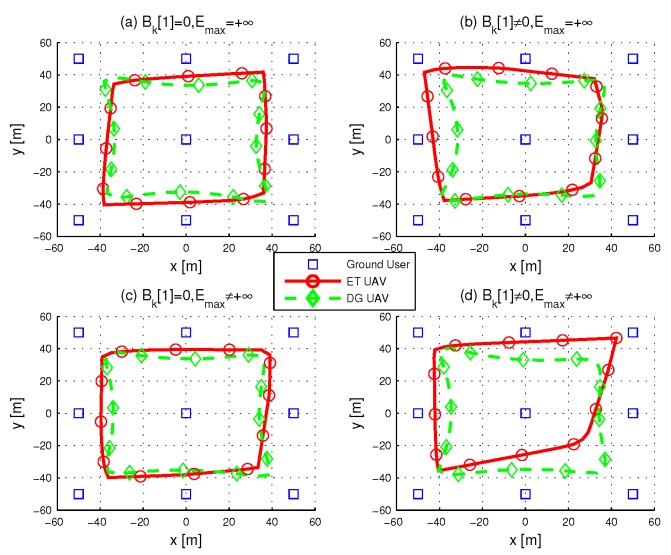
Optimized UAV trajectories when T=60s.

**Figure 5:**
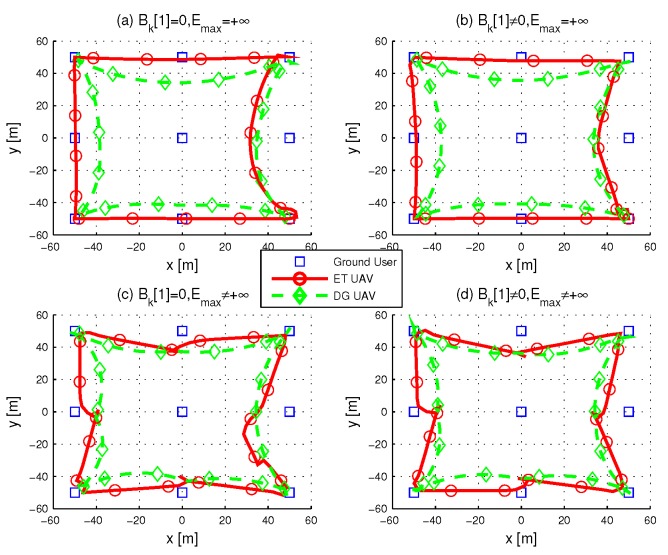
Optimized UAV trajectories when T=90s.

Figure 3, Figure 4 and Figure 5 show the optimized trajectories obtained by the proposed algorithm under different periods *T*. The total power for ET UAV is 200W. Each period considers four situations with different initial energy and energy capacity. The initial energy is unavailable for users in (a) and (c), while users in (b) and (d) have non-zero stored energy before transmission. (a) and (b) illustrate the situations with infinite users’ energy capacity, (c) and (d) shows UAVs’ trajectories when the energy capacity of users is $E_{\max}=-10\;\mathrm{dBm}$. More specifically, in (b) and (d), the initial energy of the users who are in x∈[−50,0] is less than that of the user located in x∈[0,50]. Figure 3 shows the trajectories with T=30s; it is impossible for a UAV to visit every user in such a short time period, thus both ET and DG UAV fly in the center area. (b) and (d) in Figure 3 shows that the ET UAV spends more time in left side area to charge the users whose initial energy is insufficient.

When T=60s, we can observe from (a) and (c) in Figure 4 that no matter whether energy capacity is infinite or not, both ET UAV and DG UAV tried to visit every ground user. This because only when a UAV flies over a user does it achieve the best channel gain. By comparing (a) and (c) with (b) and (d), one key observation is that the initial energy in users still plays key role in ET UAV trajectory where ET UAV does not visit users whose initial energy is sufficient. We plot the UAV trajectories in Figure 5 when T=90, and here, as *T* is sufficiently large, the initial energy in users has no significant influence on the average throughput so that both ET and DG UAV can visit every user in all situations.

**Figure 6:**
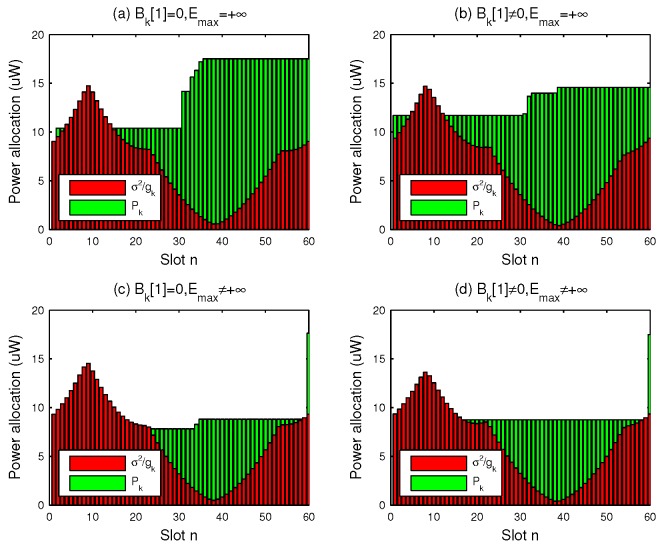
Optimal power allocation at user when T=60s.

**Figure 7:**
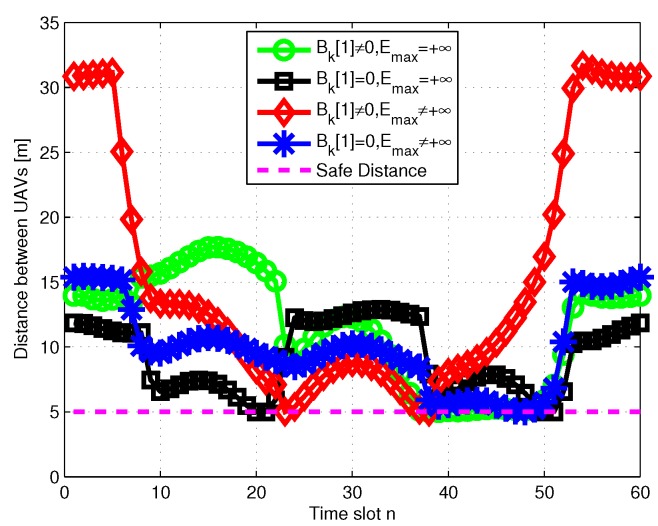
Distance between UAVs when T=60s.

**Figure 8:**
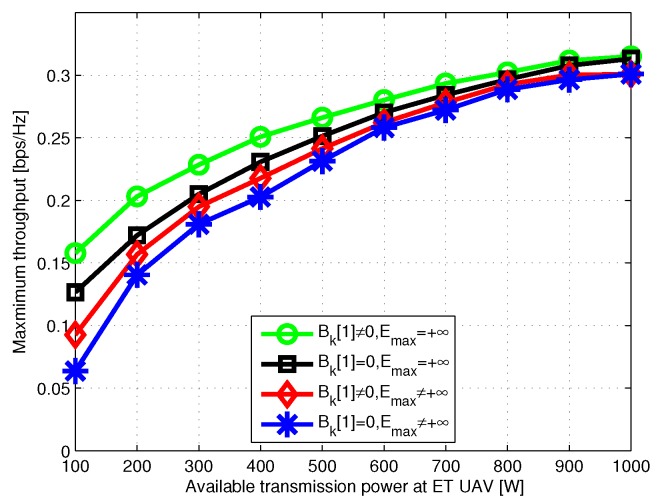
Minimum throughput versus transmission power at ET UAV when T=60s.

In Figure 6, the power allocation to the user located in the southwest of the area is plotted. It is observed that in all cases the optimal power allocation performs staircase “water-filling”, where the water levels are not decreased. We check the distance between the two UAVs in Figure 7, and the results show that they always fly with the safety distance maintained. Figure 8 shows the minimum throughput of the *K* user versus the available transmission power at ET UAV, with T=60s; the throughput increases more slowly when the available transmission power becomes large. Furthermore, with sufficient power at ET UAV, the performance gap is observed to become small, because the user can always transmit with their peak power.

**Figure 9:**
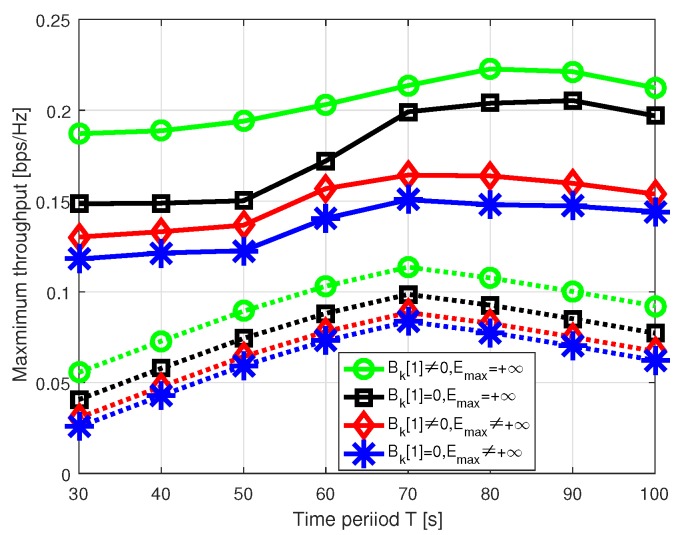
Minimum throughput versus time period *T*.

**Figure 10:**
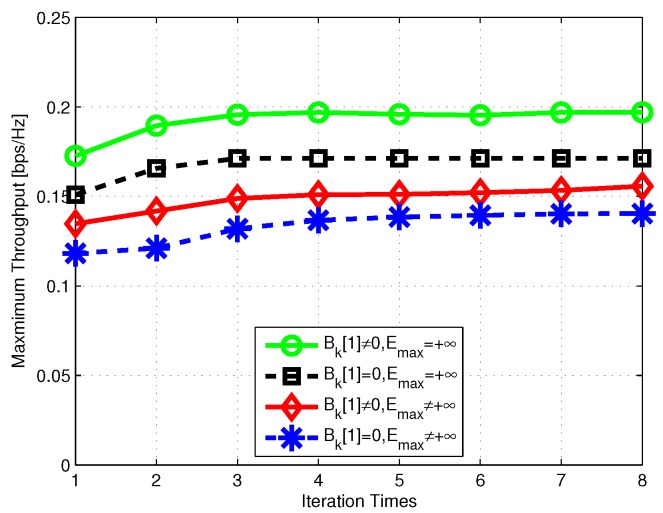
Convergence of proposed algorithm.

Now, we compare the minimum-throughput performance for different situations with respect to the time period *T* in Figure 9. To validate the effectiveness of our proposed scheme, the performance of initial trajectory without power allocation is also plotted. It is observed that our scheme can significantly improve the minimum throughput. On one hand, with the time interval *T* increased, the UAV can visit more GUs, meaning there may be more time slots that can enjoy the favorable channel gain; on the other hand, as the energy for WET is a constraint, the increased time interval may decrease the average throughput. Hence, as shown in Figure 9, the throughput of the whole system first increases then decreases with the increase of *T*. Finally, we evaluate the convergence of our proposed algorithm for T=60s; in Figure 10, the algorithm converges within at most 5 iterations for different situations.

## 5. Conclusions

In this paper, we have investigated the multi-UAV-enabled WPCN where the ET UAV is dedicated to WET and the DG UAV collects data from multiple GUs. The UAV trajectories, power allocation at the GUs, and ET UAV are jointly optimized to maximize the minimum throughput among all GUs. To solve the non-convex problem, we divide the primal problem into two subproblems. By means of alternating optimization and successive convex optimization techniques, the locally optimal solution can be efficiently obtained by the proposed algorithm, which has been shown to converge. Numerical results demonstrate the efficiency of the proposed algorithm in different scenarios.

## References

[1] Zeng Y., Zhang R., Lim T.J. (2016). Wireless communications with unmanned aerial vehicles: Opportunities and challenges. IEEE Commun. Mag..

[2] Lyu J., Zeng Y., Zhang R., Lim T.J. (2017). Placement optimization of UAV-mounted mobile base stations. IEEE Commun. Lett..

[3] He H., Zhang S., Zeng Y., Zhang R. (2016). Joint altitude and beamwidth optimization for UAV-enabled multiuser communications. IEEE Commun. Lett..

[4] Zeng Y., Zhang R., Lim T.J. (2016). Throughput maximization for UAV-enabled mobile relaying systems. IEEE Trans. Commun..

[5] Zhan C., Zeng Y., Zhang R. (2018). Energy-Efficient Data Collection in UAV Enabled Wireless Sensor Network. IEEE Wirel. Commun. Lett..

[6] Xu Y., Xiao L., Yang D., Wu Q., Cuthbert L. (2018). Throughput Maximization in Multi- UAV Enabled Communication Systems With Difference Consideration. IEEE Access.

[7] Yang D., Wu Q., Zeng Y., Zhang R. (2018). Energy Tradeoff in Ground-to-UAV Communication via Trajectory Design. IEEE Trans. Veh. Technol..

[8] Zhou X., Ho C.K., Zhang R. Wireless power meets energy harvesting: A joint energy allocation approach. Proceedings of the 2014 IEEE Global Conference on Signal and Information Processing (GlobalSIP).

[9] Ju H., Zhang R. (2014). Throughput maximization in wireless powered communication networks. IEEE Trans. Wirel. Commun..

[10] Energous Corp.. https://www.energous.com/.

[11] Ossia Inc.. https://www.ossia.com/.

[12] Xu J., Zeng Y., Zhang R. (2018). UAV-Enabled Wireless Power Transfer: Trajectory Design and Energy Optimization. IEEE Trans. Wirel. Commun..

[13] Xie L., Xu J., Zhang R. (2018). Throughput Maximization for UAV-Enabled Wireless Powered Communication Networks. IEEE Internet Things J..

[14] Park J., Lee H., Eom S., Lee I. (2018). Minimum Throughput Maximization in UAV-Aided Wireless Powered Communication Networks. https://arxiv.org/abs/1801.02781.

[15] Wu Q., Zeng Y., Zhang R. (2018). Joint trajectory and communication design for multi-UAV enabled wireless networks. IEEE Trans. Wirel. Commun..

[16] Boyd S., Vandenberghe L. (2004). Convex Optimization.

[17] Grant M., Boyd S. (2014). CVX: Matlab Software for Disciplined Convex Programming, Version 2.1. http://cvxr.com/cvx.

